# Anticoagulation therapy and clinical outcomes following transcatheter mitral valve repair for patients with mitral regurgitation: A meta‐analysis

**DOI:** 10.1002/clc.24017

**Published:** 2023-04-10

**Authors:** Jian Zhang, Yu Yang, Lin Jia, Jiannan Su, Ai Xiao, Xianhe Lin

**Affiliations:** ^1^ Cardiology Department The First Affiliated Hospital of Anhui Medical University Hefei Anhui China; ^2^ Graduate School Anhui Medical University Anhui China

**Keywords:** anticoagulants, mitral regurgitation, transcatheter mitral valve repair

## Abstract

Transcatheter mitral valve repair (TMVR) using MitraClip (MC) is now an established technique in the interventional treatment of mitral regurgitation. Common complications after MC procedure are bleeding and ischemic events. However, 2017 ESC/EACTS and 2020 ACC/AHA did not give a clear antithrombotic protocol, the policy has been based on clinical experience. Here, we performed a meta‐analysis comparing outcomes with and without the addition of anticoagulants after TMVR. We searched the Cochrane Library, EMBASE, PubMed, and Web of Science from inception to October 6, 2022 to identify studies with or without the use of anticoagulants after TMVR. From each study, we extracted the number of people with bleeding, stroke, combined endpoints, and all‐cause death. Five observational cohort studies were included, enrolling a total of 1892 patients undergoing TMVR who were assigned to either the anticoagulation group (*n* = 1209) or the no‐anticoagulation group (*n* = 683). Pooled analysis showed a significantly lower stroke rate in the anticoagulated group (at least 4 weeks duration) compared with the non‐anticoagulated group (RR [95% CI] = 0.14 [0.0−0.77], *p* = 0.02), and similar rates of bleeding, combined endpoints, and all‐cause death in both groups (RR [95% CI] = 0.76 [0.48−1.22], *p* = 0.26), (RR [95% CI] = 0.52 [0.10−2.63], *p* = 0.43), and (RR [95% CI] = 0.89 [0.58−1.35], *p* = 0.58). We observed a reduced risk of stroke without elevated risk of bleeding, combined endpoints, or all‐cause death in patients using anticoagulants (at least 4 weeks duration) after TMVR compared to no anticoagulants.

## Graphical abstract

Pooled analysis showed a significantly lower stroke rate in the intervention group (anticoagulated group) compared with the control group (non‐anticoagulated group) (RR [95% CI] = 0.14 [0.02−0.77], *p* = 0.02), and similar rates of bleeding, combined endpoints, and all‐cause death in both groups (RR [95% CI] = 0.76 [0.48−1.22], *p* = 0.26), (RR [95% CI] = 0.52[0.10−2.63], *p* = 0.43), and (RR [95% CI] = 0.89 [0.58−1.35], *p* = 0.58).

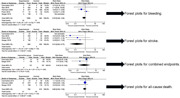

## 1 INTRODUCTION

The mitral valve, also known as the left atrioventricular valve, is attached to the left fibrous atrioventricular ring and is formed by folds of the endocardium. Mitral valve disease mainly includes mitral regurgitation, mitral stenosis, and mitral valve prolapse, with mitral regurgitation accounting for the majority of all mitral valve disease and even affecting more than 10% of the population over 75 years of age.^1^

Severe mitral regurgitation that affects the quality of life requires aggressive treatment. Pharmacological treatment only improves symptoms and does not improve survival.^2^ In the absence of surgical intervention, patients with severe mitral regurgitation have an annual mortality rate of 5%^3^, ^4^ and a 5‐year mortality rate of up to 60% when combined with severe heart failure.^5^, ^6^ Therefore, if the situation permits, surgical treatment is recommended,^7^ it improves survival, morbidity, and the quality of life. However, patients treated with mitral valve repair or replacement often exhibit advanced age, frailty, and substantial complications and are considered to be at high risk for or unable to tolerate the procedure, and transcatheter mitral valve repair (TMVR) emerged as an alternative treatment. Over the past two decades, TMVR using the MitraClip (MC) system has been increasingly and successfully used to treat degenerative and functional mitral regurgitation.^8^ As TMVR causes endothelial damage and the implanted MC increases the risk of thrombosis until endothelialization is complete, this process carries the risk of thromboembolic stroke and requires intensive anticoagulation to prevent thrombosis. Cases of postoperative left atrial or left ventricular thrombosis have been reported.^9^, ^10^ However, patients often exhibit risk factors for bleeding such as congestive heart failure, hypertension, advanced age, renal insufficiency, and diabetes mellitus. ESC/EACTS 2017 and ACC/AHA 2020 did not give a clear antithrombotic protocol,^11^ current policy is largely dependent on the experience of the clinician. Here, we conducted a meta‐analysis of clinical studies investigating the clinical outcomes of patients receiving TMVR with or without anticoagulant therapy, drawing conclusions that we expect will help clinicians' decisions.

## 2 MATERIALS AND METHODS

### 2.1 Protocol and registration

This systematic review was performed according to the Preferred Reporting Items for Systematic Reviews and Meta‐Analyses (PRISMA) criteria,^12^ and registered with PROSPERO (International Prospective Register of Systematic Reviews, CRD42022369486).

### 2.2 Search strategy and source

Two broad search terms (“TMVR” and “antithrombotic”) were used and combined using the Boolean operator “AND.” For the search term “TMVR,” the combination of the following text words, entry terms, and Mesh were used: TMVR, transcatheter mitral valve repair, percutaneous mitral valve repair, MitraClip, mitral clip, mitral valve transcatheter edge‐to‐edge repair. For the search theme “antithrombotic,” the combination of the following text words was used: antiplatelet, antithrombotic, anticoagulation, anticoagulant, aspirin, clopidogrel, P2Y receptor antagonist, edoxaban, apixaban, rivaroxaban, dabigatran, vitamin K antagonist, non‐vitamin K oral anticoagulant. The above keywords were used and combined using the Boolean operator “OR.” The references listed in all eligible studies were checked to identify further citations.

### 2.3 Study selection and screening criteria

The following selection and screening criteria were used to perform our study according to the PICOS principles. Participants (P): Patients with TMVR. Intervention (I): anticoagulation therapy. Comparison (C): without anticoagulation therapy. Outcomes (O): bleeding, stroke, combined endpoints, and all‐cause death. Study design (S): observational and experimental studies. Reviews, meta‐analysis, uncompleted clinical trials, and studies involving patients who did not receive anticoagulant treatment were excluded. Two investigators (Y. Y. and L. J.) independently assessed records for eligibility by title and abstract and the other two (J. N. S. and A. X.) by full text. Disagreements were resolved by a third investigator (J. Z.).

### 2.4 Data extraction

Two reviewers (J. N. S. and A. X.) independently extracted and recorded all data using a standard form. Data were checked by a third reviewer (J. Z.). Information extracted included: first author, year of publication, country, study design, duration of follow‐up, total number of patients, age, sex, number of patients per intervention, outcomes (bleeding, stroke, combined endpoints and all‐cause death, the combined endpoints being a combination of all‐cause death, all strokes, all bleeding), hypertension, diabetes, previous myocardial infarction, peripheral vascular disease, chronic kidney disease, chronic obstructive lung disease, heart failure, and so forth.

### 2.5 Quality assessment standard

Two reviewers (Y. Y. and L. J.) independently assessed the risk of bias of individual studies with the Newcastle−Ottawa scale (NOS) for studies. Disagreements were resolved by a third reviewer (J. Z.). With the NOS, scores <4 indicate a high risk of bias, scores between 4 and 6 indicate an intermediate risk of bias, and scores of 7 or higher indicate a low risk of bias.

### 2.6 Statistical analysis

Review Manager version 5.4 and Stata 17 were used to conduct this analysis. The means of continuous variables and the frequencies of categorical variables were extracted from baseline features of participants enrolled in each included study. RR (risk ratio) and its 95% CI (confidence intervals) were calculated to combinate the categorical variable data in this meta‐analysis. Among combined study results, the degree of inconsistency (*I* ^2^) were used to assess the heterogeneity. *I* ^2^ values of approximately 25%, 50%, and 75% were considered to indicate low, moderate, or high heterogeneity, respectively. A Mantel–Haenszel fixed‐effects model was used when *I* ^2^ was less than 50%. Otherwise, the Mantel–Haenszel random‐effects model was used. *p* < 0.05 indicated statistical significance. We also performed a subgroup analysis stratifying study design, according to country. Forest plots were generated to show the relative effect size for each clinical outcome. Publication bias was assessed by examining funnel plot asymmetry and Egger's regression test. Sensitivity analysis was used to judge the stability of the ultimate results.

## 3 RESULTS

### 3.1 Search results

**Figure 1:**
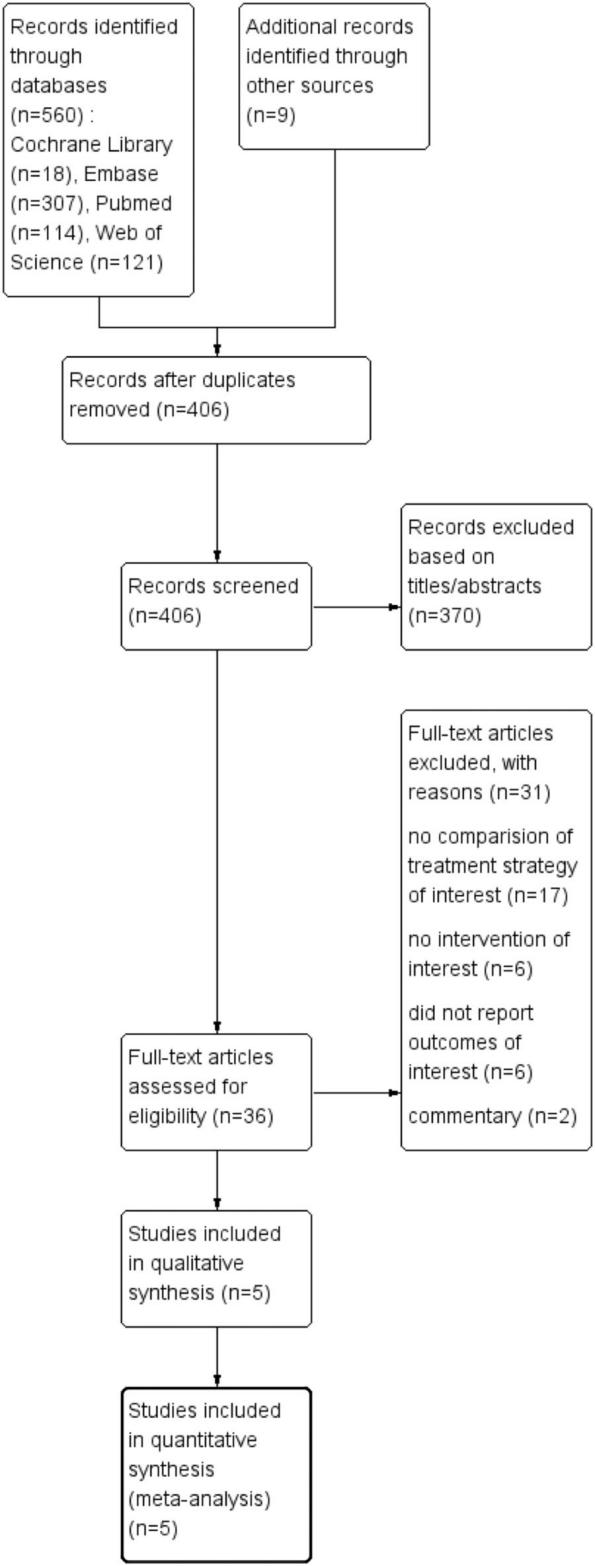
Flow chart of literature screening and the selection process.

After screening 569 articles, 5 studies published before October 6, 2022, were included in this meta‐analysis. The flow chart of the literature screening and the selection process is shown in Figure 1.^13^, ^14^, ^15^, ^16^, ^17^

### 3.2 Characteristics of eligible studies

Treatment allocation was open‐label in all cases. Five studies were observational cohort studies, including 1892 patients (1209 patients in anticoagulation group and 683 patients in no‐anticoagulation group), and investigation sites were located in Germany and Italy. The main characteristics of included research are shown Table 1. In brief, all studies enrolled patients with moderate to severe mitral regurgitation treated with TMVR. The treatment regimen in the control group did not use anticoagulants but SAPT or DAPT. The treatment regimen in the intervention group used anticoagulants, with or without the addition of antiplatelet agents, mainly OAC, except for the use of low molecular weight heparin in some patients in the Geis study.^13^

**Table 1:** Baseline characteristics of included studies.

Study					
Variable	**Seeger**	**Polzin**	**Hohmann**	**Cammalleri**	**Geis**
Year	2019	2015	2022	2014	2019
Country	Germany	Germany	Germany	Italy	Germany
Study design	A prospective observational study	An observational single‐center cohort study	A non‐interventional retrospective cohort study	A single‐center cohort study	A single‐center retrospective cohort study
Follow‐up duration	30 days, 12 months	6 months	30 days, 6 months	30 days, 12 months	30 days
Patient (*n*)	254	73	1342	60	470
Male (*n*)	N/A, similar between groups	49	841	42	277
Ages (years)	N/A, apixaban group were y younger	73 ± 10	76.4 ± 8.8	73 ± 9	75.5 ± 10.8
Heart failure (*n*)	N/A, similar between groups	51	1046	N/A	470
CHA2DS2‐VASc scores	N/A, similar between groups	N/A	4.6 ± 1.4	N/A	N/A
HAS‐BLED scores	N/A, similar between groups	N/A	3.1 ± 1.1	N/A	2.1 ± 0.3
Cause of mitral regurgitation (functional/degenerative) (*n*)	N/A	15/58	N/A	47/13	300/170
Acute procedural success (*n*)	254	73	1342	60	470
Number of clips (*n*)	N/A	N/A	N/A	One clip (*n* = 23), two clips (*n* = 36), three clips (*n* = 1)	N/A
Chronic kidney disease (*n*)	N/A, similar between groups	35	681	N/A	54
Coronary artery disease (*n*)	N/A, similar between groups	51	978	N/A	N/A
Prior myocardial infarction (*n*)	N/A, similar between groups	38	163	N/A	N/A
Previous aortocoronary bypass grafting (*n*)	N/A, similar between groups	18	N/A	N/A	N/A
Previous percutaneous coronary intervention (*n*)	N/A, similar between groups	36	24	17	N/A
Hypertension (*n*)	N/A, similar between groups	71	1218	N/A	401
Hypercholesterolemia (*n*)	N/A, similar between groups	37	N/A	N/A	N/A
Diabetes mellitus (*n*)	N/A, similar between groups	23	604	N/A	160
COPD (*n*)	N/A, similar between groups	17	N/A	N/A	N/A
History of ischemic stroke (*n*)	N/A	N/A	89	N/A	56
Control group (without anticoagulation [*n*])	118	32	458	39	36
Specific programs	SAPT (*n* = 85), DAPT (*n* = 33)	DAPT	SAPT (*n* = 301), DAPT (*n* = 157)	SAPT (*n* = 22), DAPT (*n* = 17)	SAPT (*n* = 4), DAPT (*n* = 32)
Intervention group (with anticoagulation [*n*])	136	41	577	21	434
Specific programs	Apixaban plus ASA for 4 weeks, then ASA alone	DAPT plus OAC for at least 6 months	OAC (*n* = 261), OAC plus SAPT (*n* = 279), OAC plus DAPT (*n* = 37) (anticoagulation for at least 6 months)	OAC (*n* = 6), OAC plus SAPT (*n* = 15) (anticoagulation for at least 12 months)	OAC (*n* = 298), OAC plus DAPT (*n* = 97), low molecular weight heparin (*n* = 39) (anticoagulation for at least 30 days)
Arteriosclerosis (*n*)	N/A	N/A	293	N/A	N/A
History of any bleeding event (*n*)	N/A	N/A	241	N/A	14
Atrial fibrillation (*n*)	N/A, similar between groups	N/A	838	N/A	289
Previous venous thromboembolism (*n*)	N/A	N/A	53	N/A	N/A

Abbreviations: APT, antiplatelet therapy; ASA, aspirin; COPD, chronic obstructive lung disease; DAPT, dual antiplatelet therapy; OAC, oral anticoagulation; SAPT, single antiplatelet therapy.

### 3.3 Quality assessments

The quality of cohort studies was determined according to the NOS scoring system and quality scores all varied from 7 to 8, indicate a low risk of bias. Specific data are shown in Supporting Information: Table 1.

### 3.4 Data analysis, subgroup analysis

**Figure 2:**
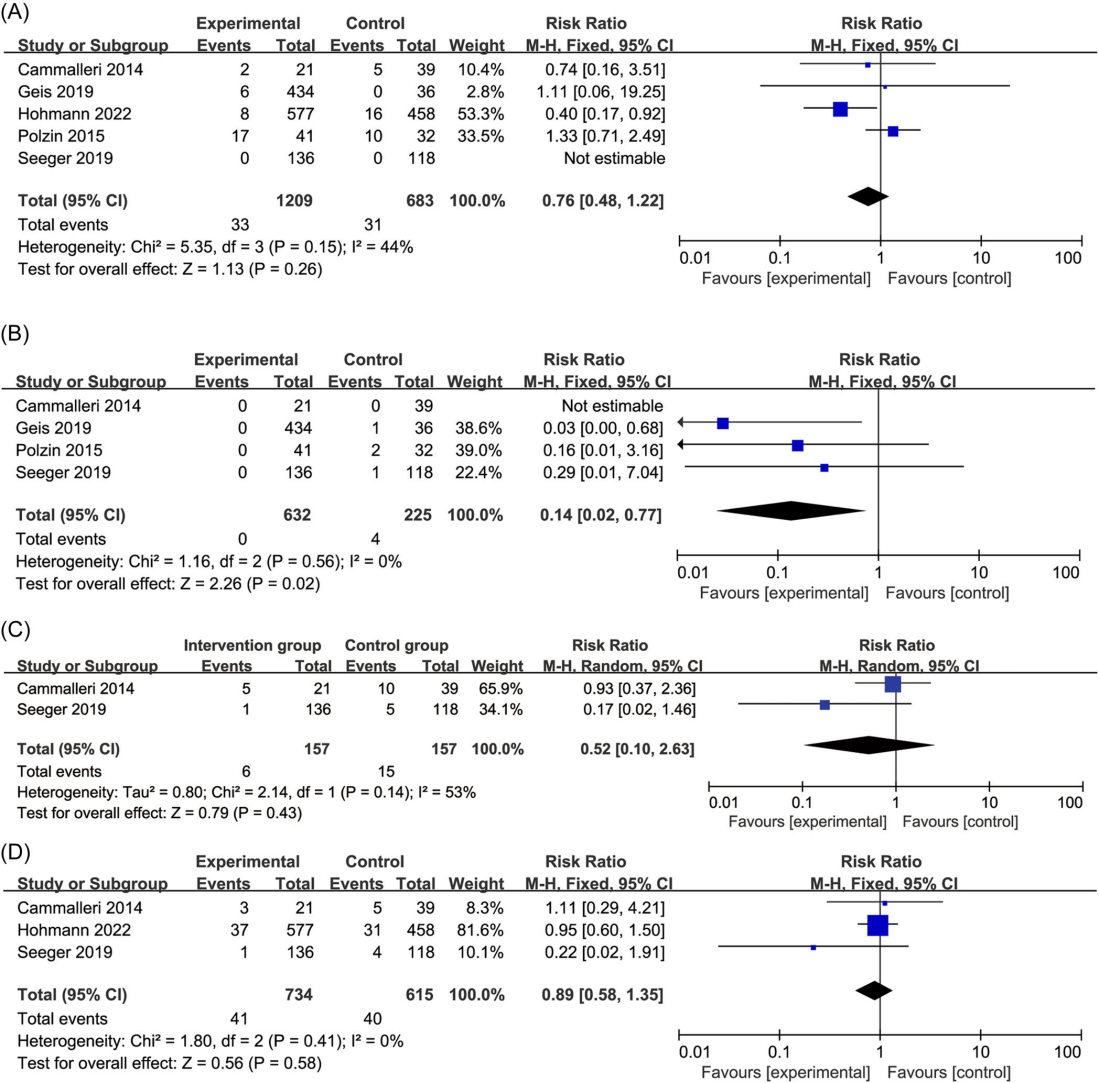
Forest plots for bleeding (A), stroke (B), combined endpoints (C), and all‐cause death (D).

All outcomes were presented in Supporting Information: Table 2. During the follow‐up period, there were 33 cases of bleeding in the intervention group and 31 cases in the control group. There was no statistically significant difference in the incidence between the two groups (RR = 0.76, 95% CI: [0.48−1.22], *p* = 0.26), with moderate heterogeneity (*I* ^2^ = 44%) (details available in Figure 2A).

There were 0 cases of stroke in the intervention group and 4 cases in the control group. There was a statistically significant difference in the incidence between the two groups (RR = 0.14, 95% CI: [0.02−0.77], *p* = 0.02), with low heterogeneity (*I* ^2^ = 0%) (details available in Figure 2B).

There were 6 cases of combined endpoints in the intervention group and 15 cases in the control group. There was no statistically significant difference in the incidence between the two groups (RR = 0.52, 95% CI: [0.10−2.63], *p* = 0.43), with high heterogeneity (*I* ^2^ = 53%) (details available in Figure 2C).

There were 41 cases of all‐cause death in the intervention group and 40 cases in the control group. There was no statistically significant difference in the incidence between the two groups (RR = 0.89, 95% CI: [0.58−1.35], *p* = 0.58), with low heterogeneity (*I* ^2^ = 0%) (details available in Figure 2D).

The subgroups of the intervention group (anticoagulant group) were OAC, OAC plus SAPT, and OAC plus DAPT, and they were compared with the control group (non‐anticoagulant group) in terms of bleeding, stroke, combined endpoints, and all‐cause death, respectively. During the follow‐up period, there were 3 cases of bleeding in the OAC group and 5 cases in the control group. There was no statistically significant difference in the incidence between the two groups (RR = 0.64, 95% CI: [0.09−4.84], *p* = 0.67), with low heterogeneity (*I* ^2^ = 0%). There were 2 cases of bleeding in the OAC plus SAPT group and 5 cases in the control group. There was no statistically significant difference in the incidence between the two groups (RR = 1.04, 95% CI: [0.23−4.79], *p* = 0.96), heterogeneity: not applicable. There were 18 cases of bleeding in the OAC plus DAPT group and 10 cases in the control group. There was no statistically significant difference in the incidence between the two groups (RR = 1.32, 95% CI: [0.71−2.45], *p* = 0.39), with low heterogeneity (*I* ^2^ = 0%) (details available in Supporting Information: Figure 1A).

There were 0 case of stroke in the OAC group and 1 case in the control group. There was no statistically significant difference in the incidence between the two groups (RR = 0.04, 95% CI: [0.00−0.99], *p* = 0.05), heterogeneity: not applicable. There were 0 case of bleeding in the OAC plus SAPT group and 1 case in the control group. There was no statistically significant difference in the incidence between the two groups (RR = 0.29, 95% CI: [0.01−7.04], *p* = 0.45), heterogeneity: not applicable. There were 0 case of bleeding in the OAC plus DAPT group and 3 cases in the control group. There was no statistically significant difference in the incidence between the two groups (RR = 0.14, 95% CI: [0.02−1.29], *p* = 0.08), with low heterogeneity (*I* ^2^ = 0%) (details available in Supporting Information: Figure 1B).

There were 1 case of combined endpoints in the OAC group and 10 cases in the control group. There was no statistically significant difference in the incidence between the two groups (RR = 0.65, 95% CI: [0.10−4.21], *p* = 0.65), heterogeneity: not applicable. There were 5 cases of bleeding in the OAC plus SAPT group and 15 cases in the control group. There was no statistically significant difference in the incidence between the two groups (RR = 0.54, 95% CI: [0.09−3.15], *p* = 0.49), with high heterogeneity (*I* ^2^ = 59%) (details available in Supporting Information: Figure 1C).

There were 1 case of all‐cause death in the OAC group and 5 cases in the control group. There was no statistically significant difference in the incidence between the two groups (RR = 1.30, 95% CI: [0.18−9.30], *p* = 0.79), heterogeneity: not applicable. There were 3 cases of bleeding in the OAC plus SAPT group and 9 cases in the control group. There was no statistically significant difference in the incidence between the two groups (RR = 0.54, 95% CI: [0.17−1.76], *p* = 0.31), with moderate heterogeneity (*I* ^2^ = 28%) (details available in Supporting Information: Figure 1D).

### 3.5 Sensitivity analysis and publication bias

In sensitivity analyses (details available in Supporting Information: Figure 2A,B,D), removing any single study did not change the outcomes for the rates of bleeding, stroke, and all‐cause death. Supporting Information: Figure 2C suggests that the Cammalleri study may have contributed to one of the causes of instability in the combined endpoint analysis model.

Publication bias was assessed using funnel plots (details available in Supporting Information: Figure 3A,B,C,D), Egger's test and Begg's test showed no evidence of publication bias in all the analyses (*p* = 0.799 and 1.000, 0.846 and 1.000, N/A and 1.000, 0.530 and 0.296 for bleeding, stroke, combined endpoints, all‐cause death, respectively).

## 4 DISCUSSION

This meta‐analysis demonstrated that anticoagulants (at least 4 weeks duration) following TMVR for patients reduced the rates of stroke without increasing the rates of bleeding compared with no anticoagulants. There were no differences in combined endpoints and all‐cause death between groups.

TMVR with the MC system has been a safe and effective treatment for mitral regurgitation in patients who cannot tolerate conventional surgery or are at high risk for surgery, and has been recommended by the 2020 ACC/AHA guidelines^18^ and the 2017 ESC/EACTS guidelines.^19^ Although TMVR is generally safe, there are still clinical problems with complications such as thrombus formation and bleeding, especially in the early postoperative period. Studies have shown that the main postoperative adverse events are bleeding, stroke and sepsis, with the incidence of stroke within 30 days after surgery ranging from 0.7% to 2.6%,^20^, ^21^, ^22^, ^23^, ^24^ the incidence of bleeding complications is about 13%.^25^, ^26^

Patients with TMVR are at high risk for thrombosis. The three elements of thrombosis, known as the Virchow's triad, include: blood stasis, endothelial injury or vessel wall injury, and hypercoagulability. Patients with mitral regurgitation have an increased prevalence of atrial fibrillation, with an incidence of up to 63%,^27^ and then significant intra‐atrial blood stasis occurs in atrial fibrillation.^28^ On the other hand, after TMVR, approximately 25% of patients show an increased mitral pressure gradient, indicating mild to moderate mitral stenosis,^29^, ^30^, ^31^ which also affects hemodynamics. For endothelial injury or vessel wall injury, the TMVR using the MC system enters the heart through the femoral vein via a delivery catheter through the inferior vena cava and the atrial septum, captures the mitral valve, adjusts it to the optimal position, and then clamps it, thereby reducing the orifice area. Therefore, damage to the endothelium and endocardium is inevitable during the operation. For hypercoagulability, Patients undergoing TMVR are mostly elderly, have multiple chronic diseases, and the procedure is considered somewhat “traumatic,” which can lead to changes in blood composition and resulting in a hypercoagulable state. The risk of bleeding is also very high in patients receiving TMVR. Because, this population is characterized by advanced age, combination of multiple chronic diseases (e.g., hypertension, hepatic insufficiency, renal insufficiency, history of stroke) and application of drugs such as antiplatelets and NSAIDs.^32^, ^33^, ^34^ So, it's hard to balance the risk between thrombosis and bleeding.

To date, there are no evidence‐based recommendations for anticoagulation after MC implantation, anticoagulation strategies are based on clinical experience or other postinterventional antithrombotic protocols, and large randomized controlled trials are lacking. Relevant pilot studies have been conducted, in the EVEREST II trial, with DAPT (aspirin and clopidogrel) for 30 days postoperatively, followed by a change to aspirin use for 5 months.^35^ In the COAPT trial, postoperative use of aspirin and/or clopidogrel was continued for at least 6 months. If OAC were available, OAC was continued without the addition of antiplatelet agents.^36^

This article summarizes the current studies on the use of anticoagulants after TMVR and finds that the use of anticoagulants did not increase the risk of bleeding compared to the non‐use of anticoagulants, but instead decreased the risk of stroke and did not increase the incidence of events such as all‐cause mortality and combined endpoints. The heterogeneity of the combined endpoints was high (53%). Sensitivity analysis revealed that it was Cammalleri's study that led to the increased heterogeneity. We believe that there are several possible reasons for this: (1) Cammalleri's study is the earliest study among the studies included in this meta‐analysis, and it is also the only study from the country of Italy, which may lead to increased heterogeneity due to the difference in national populations. (2) The number of populations included in Cammalleri's study is small, and the baseline characteristics of the populations are too few, which may have led to the lack of propensity‐matching in the study.

OAC, OAC plus SAPT, and OAC plus DAPT were compared with the non‐anticoagulated group in terms of bleeding, stroke, combined endpoints, and all‐cause death, respectively. There were no significant differences in bleeding, combined endpoints, and all‐cause death between OAC, OAC plus SAPT, and OAC plus DAPT versus the non‐anticoagulated group. However, subgroup analysis of stroke suggested that the different subgroups of the anticoagulation group were not statistically significant compared with the non‐anticoagulation group (*p* Values 0.05, 0.45, and 0.08, respectively), the difference between the anticoagulation and non‐anticoagulation groups was statistically significant for stroke (*p* = 0.02). The sample size included in the subgroup analysis was insufficient, and the results of the subgroup analysis need to be treated with caution.

We believe that in patients with a low risk of bleeding after TMVR, it is possible that the application of short‐term anticoagulants (at least 4 weeks duration) is the best therapy to avoid thrombosis and reduce the incidence of stroke and bleeding events. However, the optimal therapy (type and duration of anticoagulant drugs) still needs to be further evaluated in larger studies.

### 4.1 Limitations

Our study has several limitations. First, this analysis contains five observational cohort studies, therefore, it is subject to possible selection bias and confounding, inherent to this type of study designs. Second, the control group was receiving SAPT or DAPT, the intervention group was receiving OACs with SAPT/DAPT/ASA in some patients and only OAC in some. The intervention group was stratification by OAC, OAC plus DAPT, and OAC plus SAPT, but the sample size was small. The lack of detail in the stratification of the intervention group and control group as well as the small sample size led to low confidence in the conclusions. The finding on statistically significant lower risk of stroke is informed by a total of 4 lower stroke events. This also leads to less credible conclusion. Third, the baseline characteristics of the included population were incomplete, regarding prevalence of atrial fibrillation, risk of stroke and information on use of anticoagulants before the procedure Forth, none of the results from the included studies were proper time‐to‐event analyses. Simple measures such as RR based on time to event data and studies with variable follow‐up cannot be reliably compared.

## 5 CONCLUSION

We observed a reduced risk of stroke without elevated risk of bleeding, combined endpoints, or all‐cause death in patients using anticoagulants (at least 4 weeks duration) after TMVR compared to no anticoagulants. The included studies were small, which could explain the nonsignificant results for the bleeding and mortality endpoints. So the findings in this study are based on observational studies and have to be considered as hypothesis generating. A rigorous, well‐designed, large‐scale RCT is needed to further validate this finding and guide anticoagulant treatment strategies in this population.

## AUTHOR CONTRIBUTIONS

All authors contributed to the study conception and design. The idea for the article were performed by Jian Zhang (17610869013@163.com). The literature search and data analysis were performed by Yu Yang (1294574916@qq.com), Lin Jia (848128030@qq.com), Jiannan Su (sujiannan9013@sina.com), Ai Xiao (xiaoai9013@sina.com), and Jian Zhang. Drafted and critically revised the work were performed by Xianhe Lin and Jian Zhang.

## CONFLICT OF INTEREST STATEMENT

The authors declare no conflict of interest.

## Supporting information

Supporting information.

Supporting information.

Supporting information.

Supporting information.

Supporting information.

Supporting information.

Supporting information.

Supporting information.

Supporting information.

Supporting information.

Supporting information.

Supporting information.

Supporting information.

Supporting information.

Supporting information.

## Data Availability

The data that support the findings of this study are available from the corresponding author, [Xianhe Lin], upon reasonable request.
