# Supplementary figures and images for: Anticoagulation therapy and clinical outcomes following transcatheter mitral valve repair for patients with mitral regurgitation: A meta‐analysis
Source: Clin Cardiol. 2023 Apr 10;46(6):598–606. doi: 10.1002/clc.24017 (PMC10270264; doi:10.1002/clc.24017)

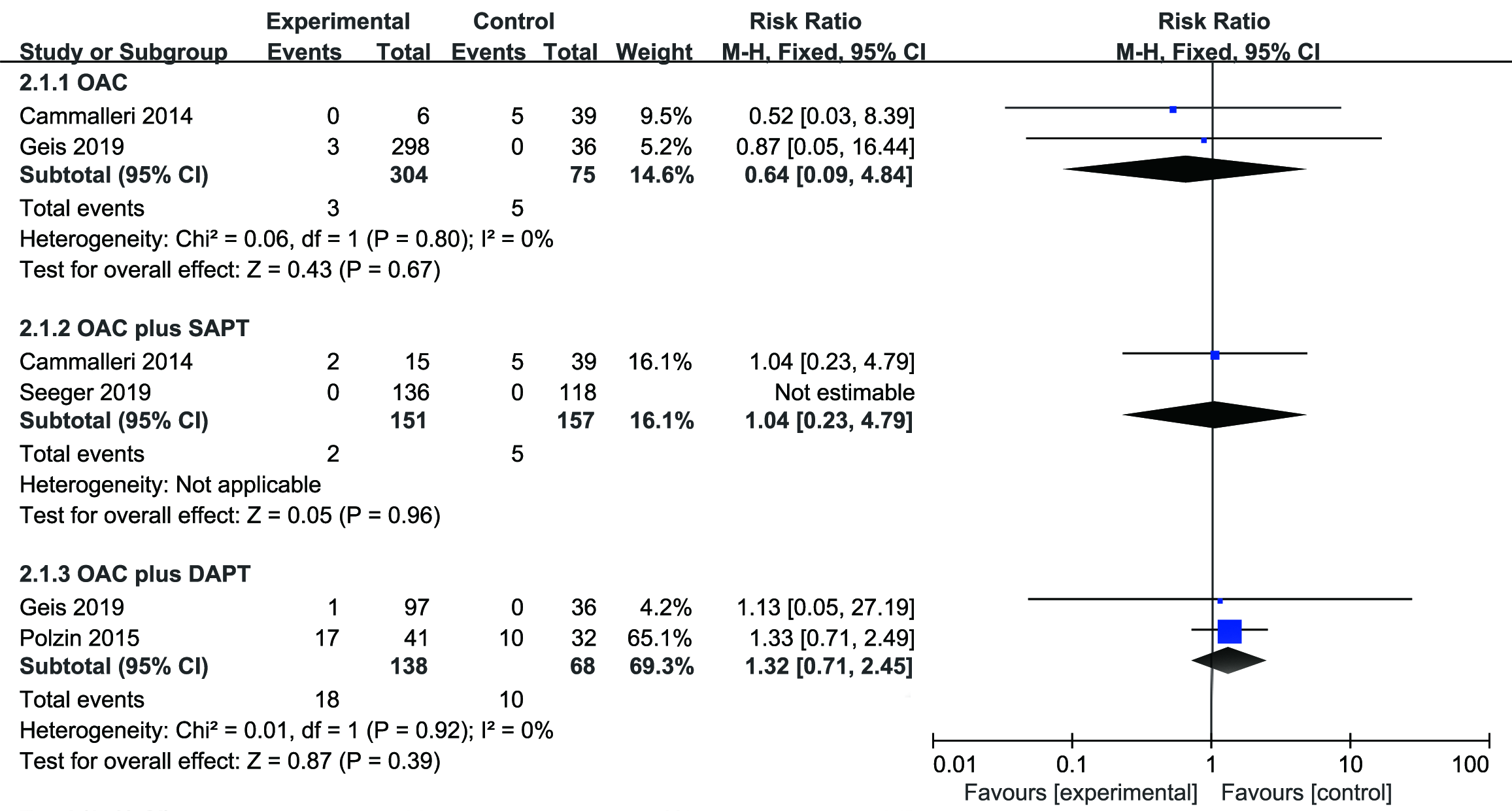

Supplement: Supplementary file 1 — Supporting information. [file CLC-46-598-s007.tif]

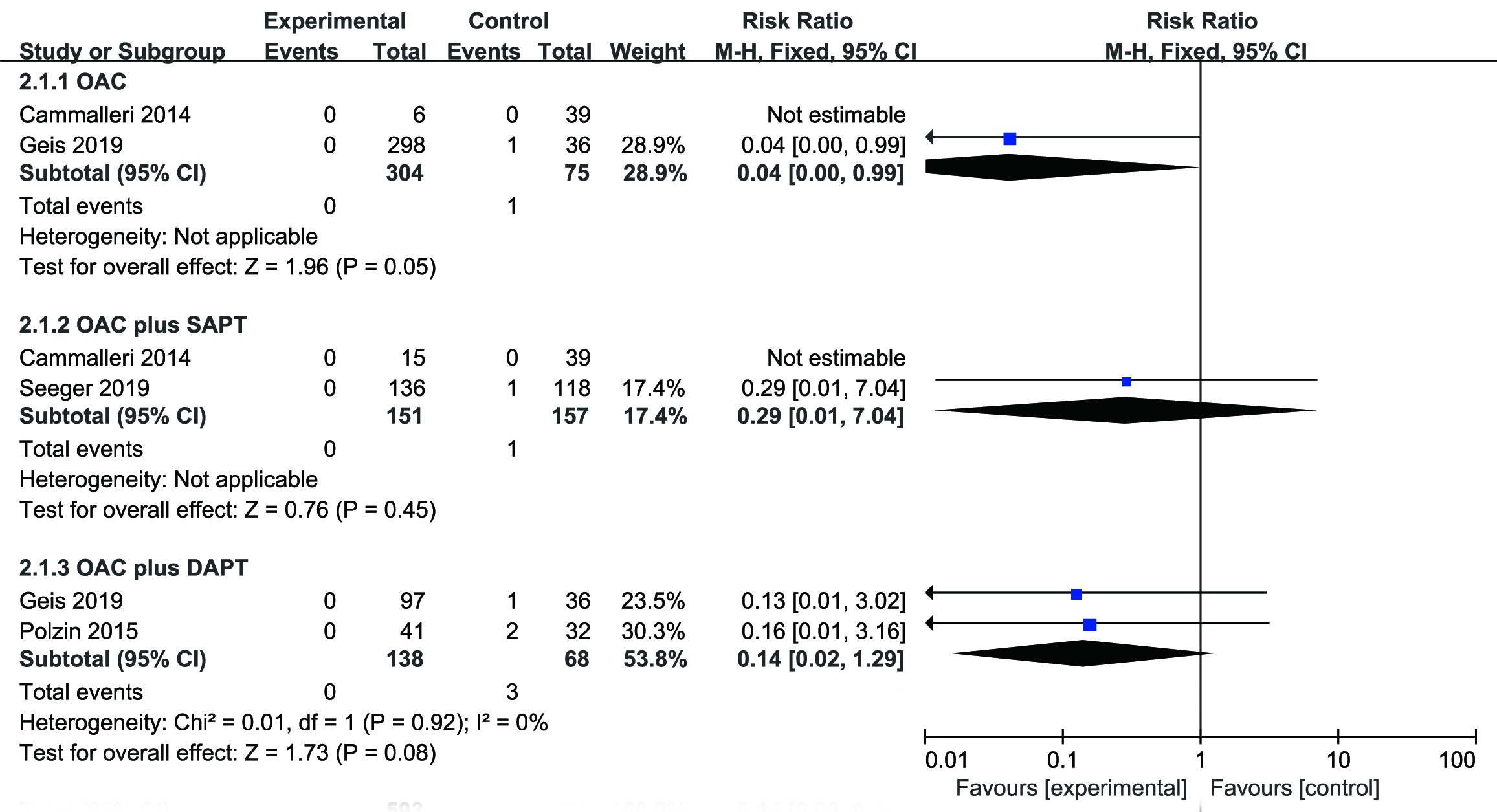

Supplement: Supplementary file 2 — Supporting information. [file CLC-46-598-s006.tif]

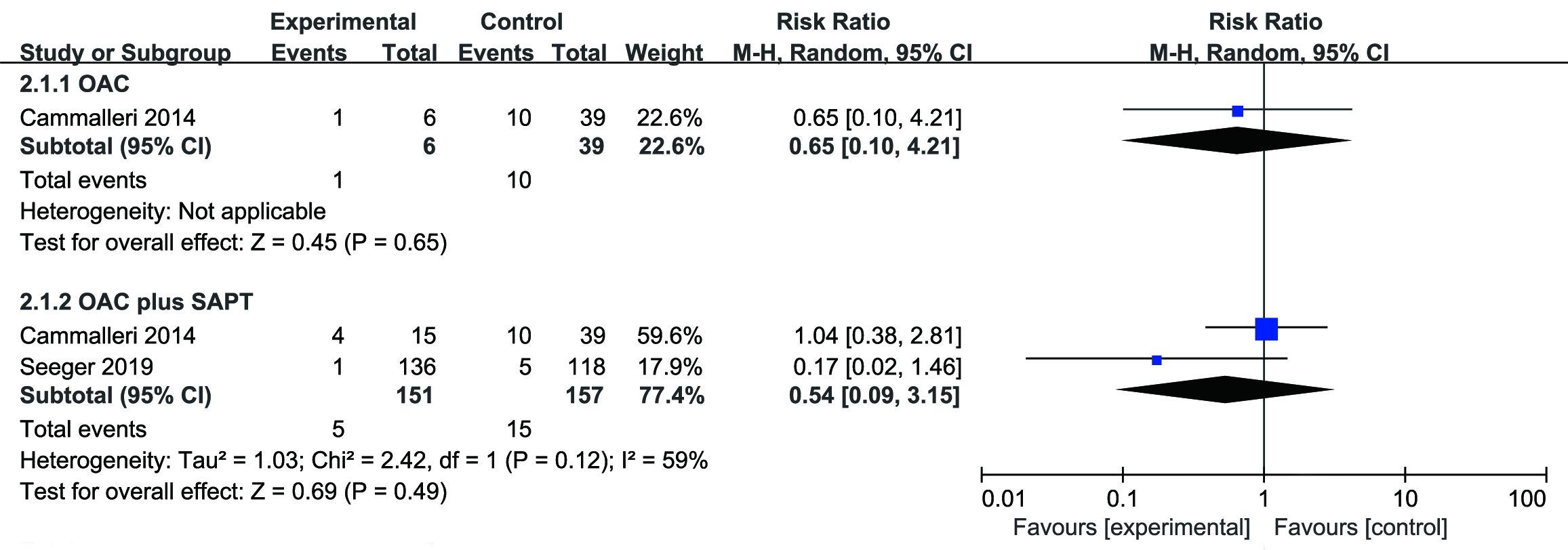

Supplement: Supplementary file 3 — Supporting information. [file CLC-46-598-s005.tif]

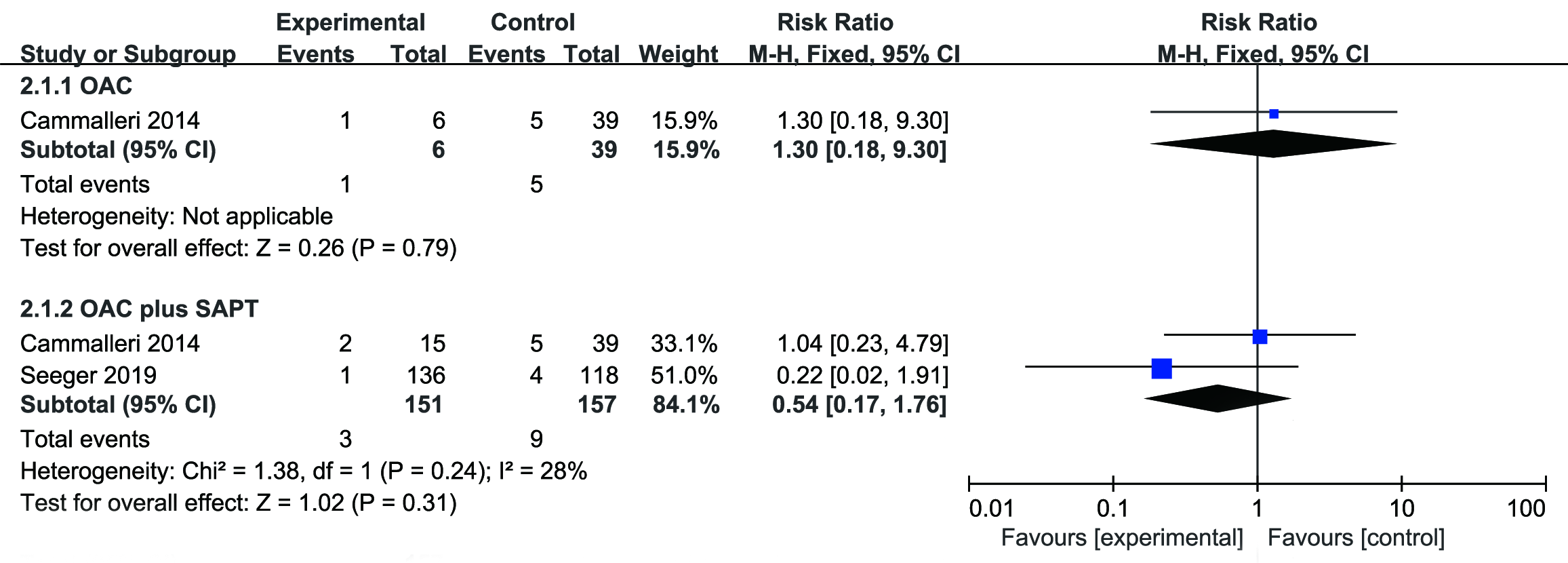

Supplement: Supplementary file 4 — Supporting information. [file CLC-46-598-s004.tif]

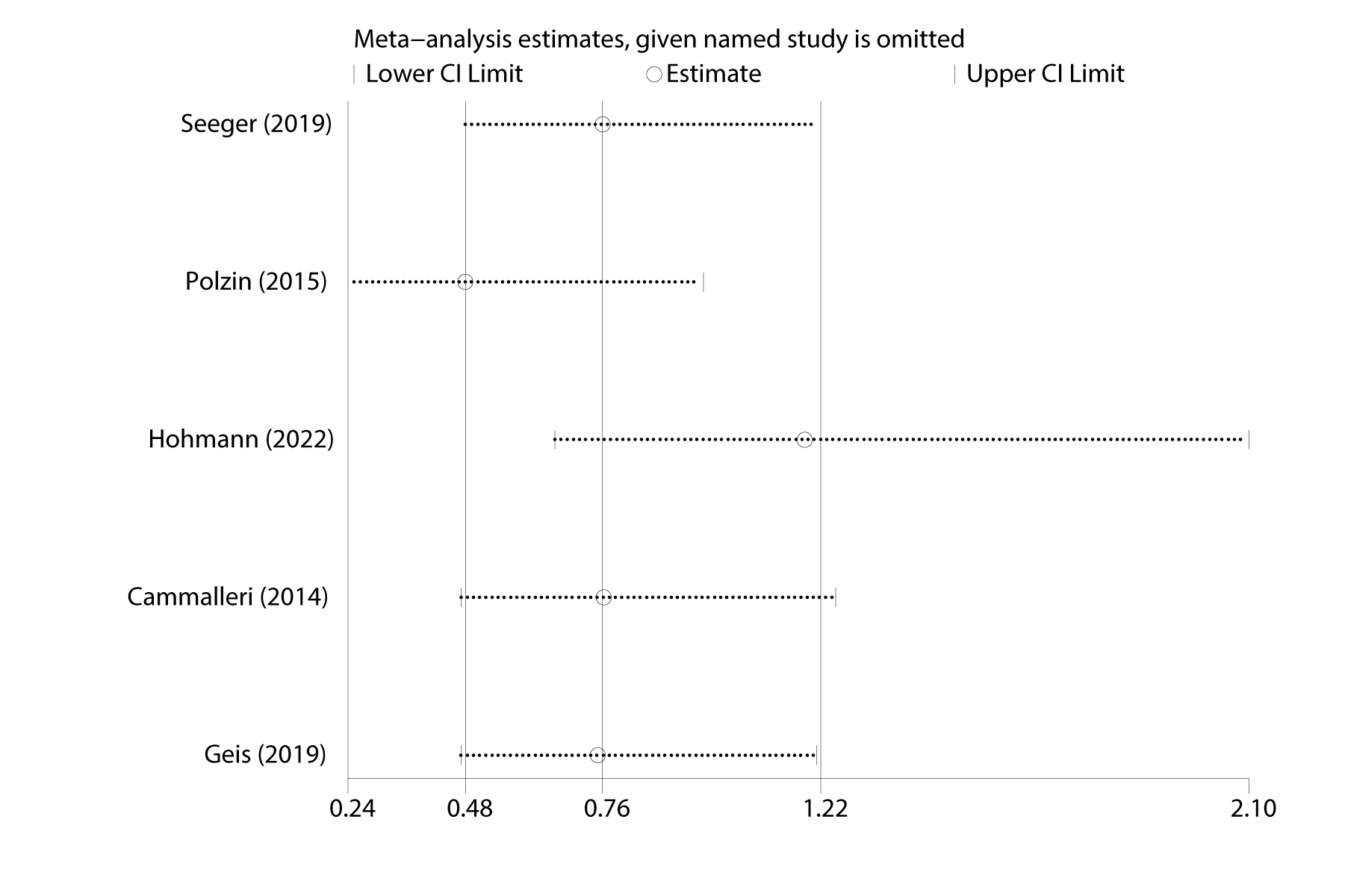

Supplement: Supplementary file 5 — Supporting information. [file CLC-46-598-s010.tif]

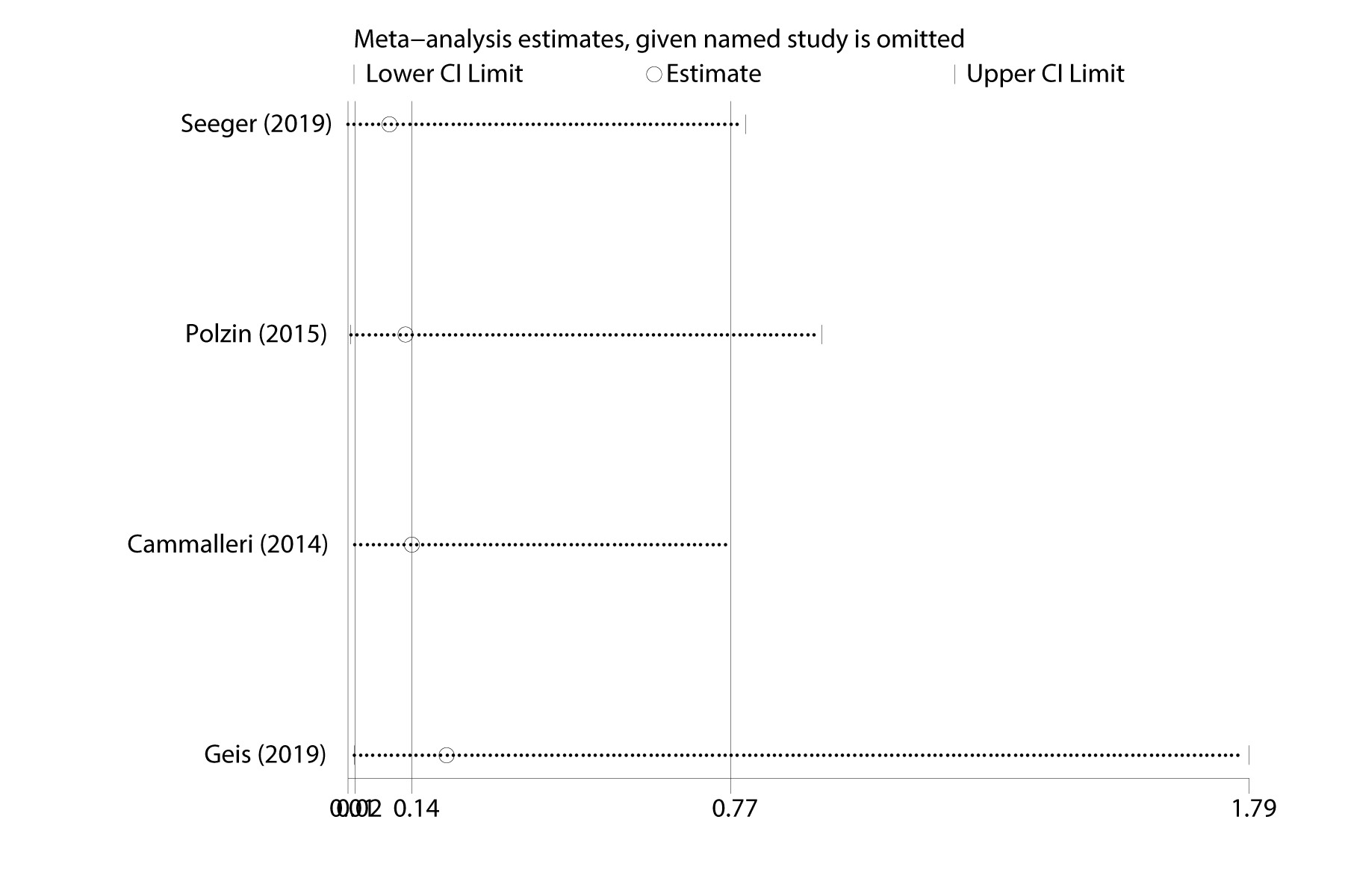

Supplement: Supplementary file 6 — Supporting information. [file CLC-46-598-s009.tif]

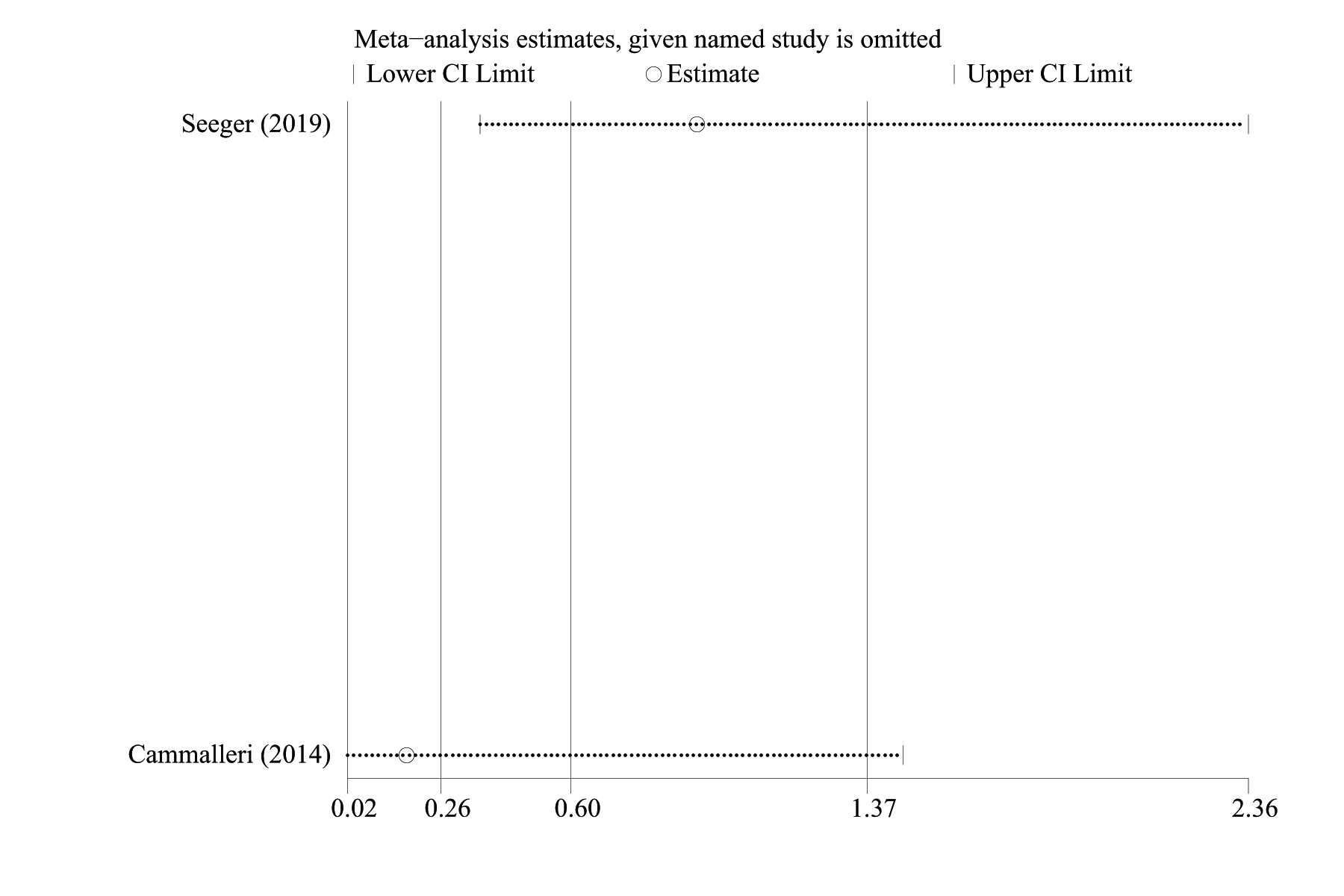

Supplement: Supplementary file 7 — Supporting information. [file CLC-46-598-s008.tif]

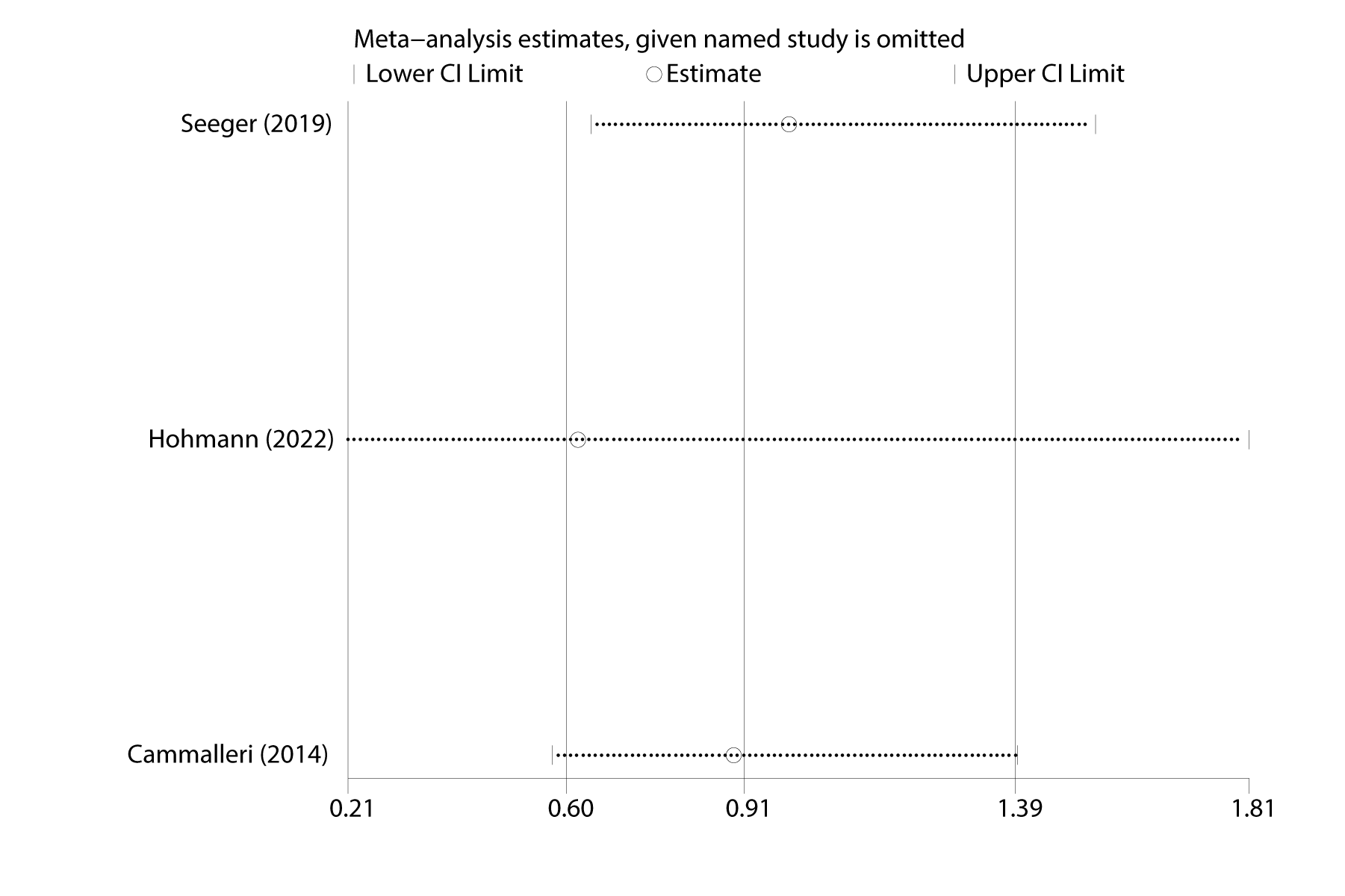

Supplement: Supplementary file 8 — Supporting information. [file CLC-46-598-s011.tif]

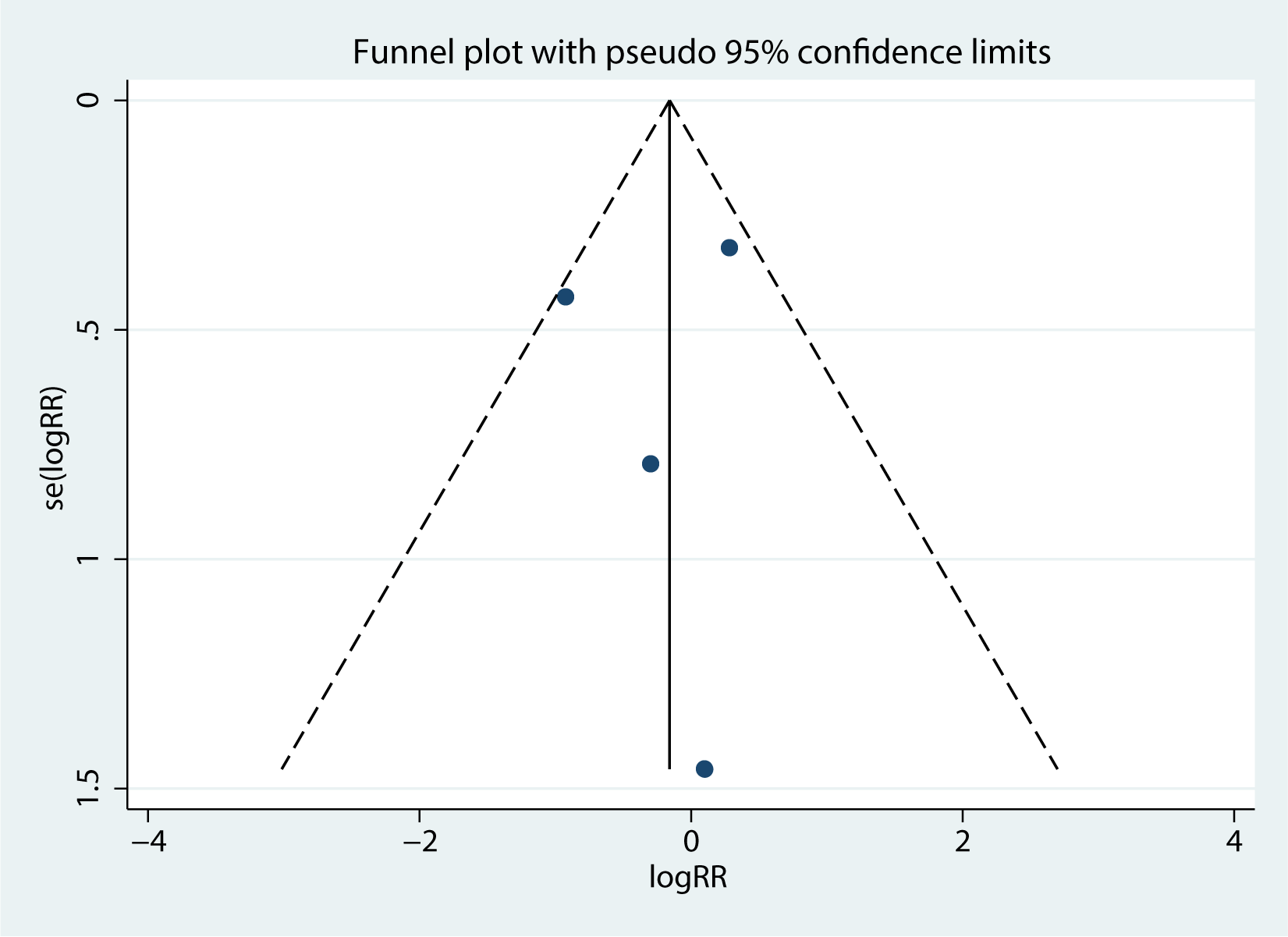

Supplement: Supplementary file 9 — Supporting information. [file CLC-46-598-s014.tif]

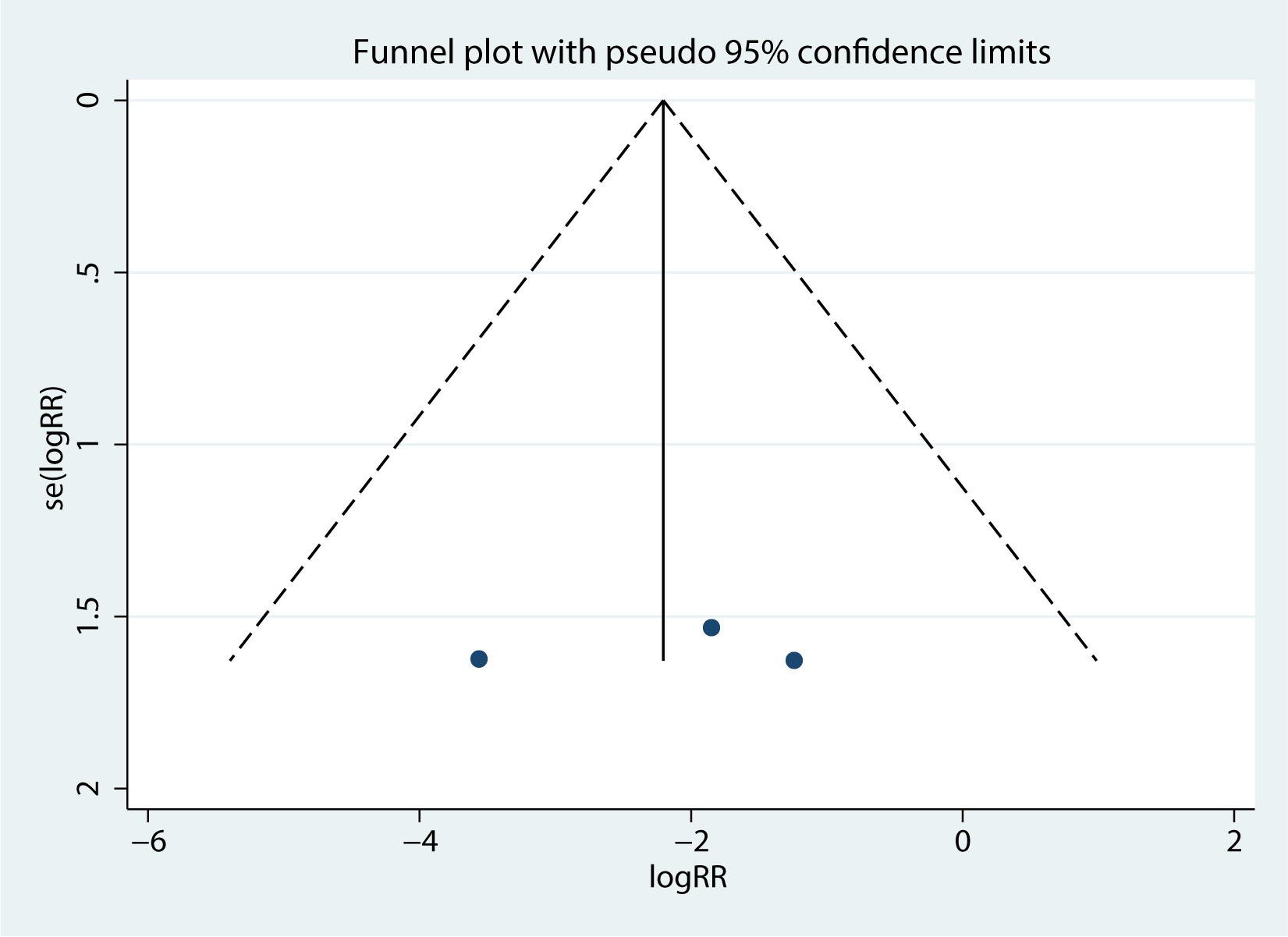

Supplement: Supplementary file 10 — Supporting information. [file CLC-46-598-s015.tif]

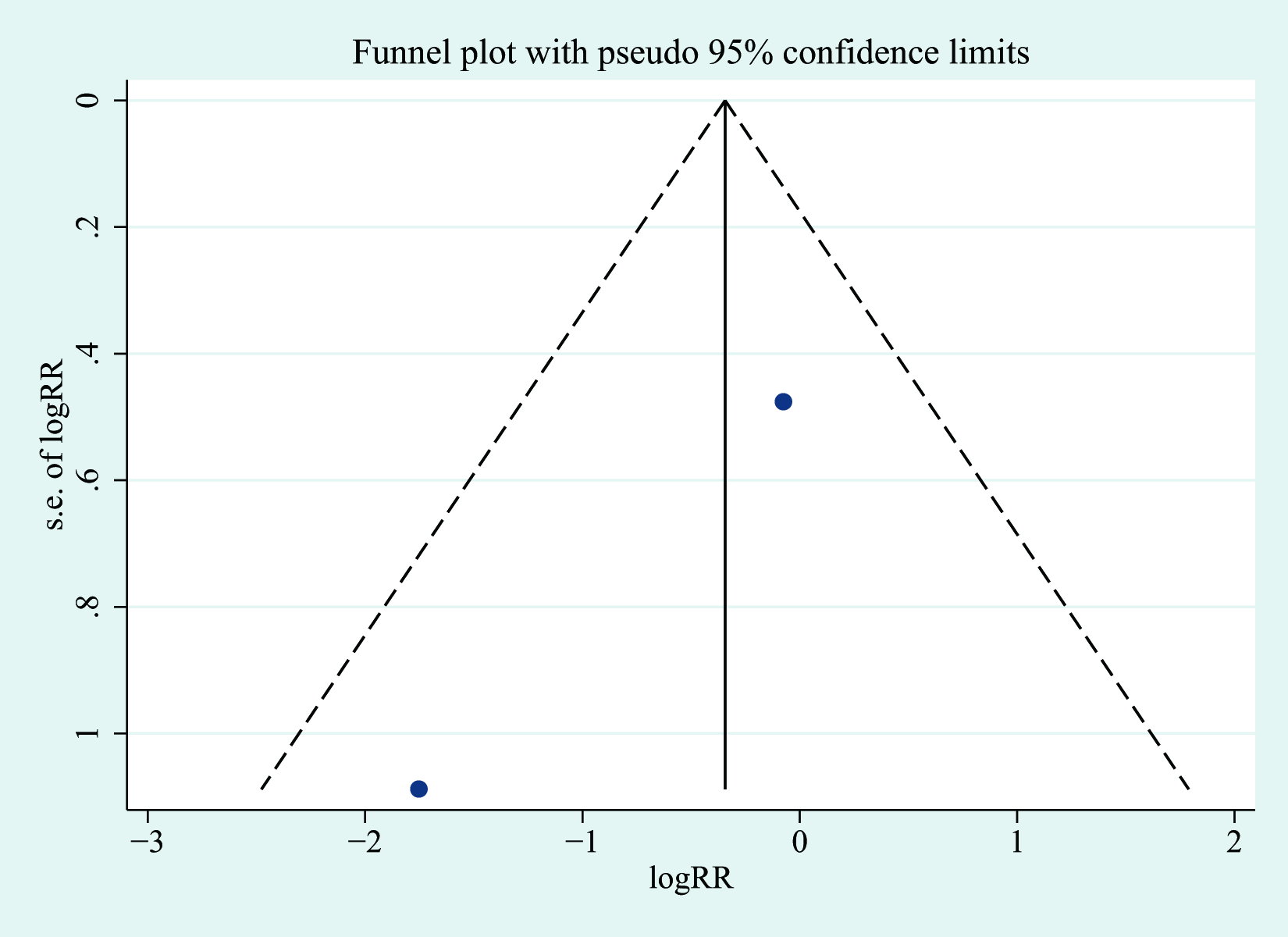

Supplement: Supplementary file 11 — Supporting information. [file CLC-46-598-s013.tif]

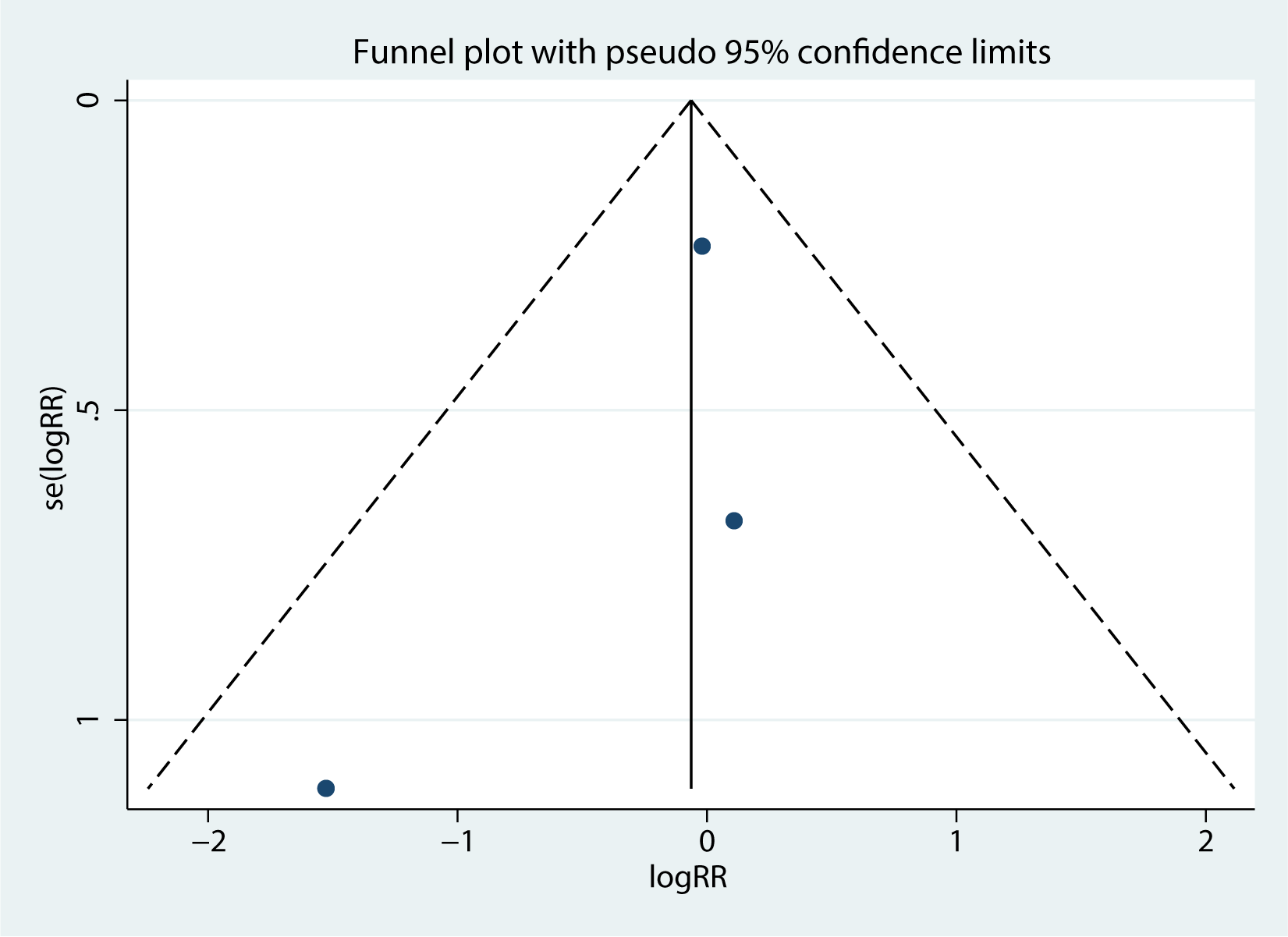

Supplement: Supplementary file 12 — Supporting information. [file CLC-46-598-s003.tif]
