# Supplementary material for: Anticoagulation therapy and clinical outcomes following transcatheter mitral valve repair for patients with mitral regurgitation: A meta‐analysis
Source: Clin Cardiol. 2023 Apr 10;46(6):598–606. doi: 10.1002/clc.24017 (PMC10270264; doi:10.1002/clc.24017)
Supplement: Supplementary file 13 — Supporting information. [file CLC-46-598-s001.docx]

| Quality assessment of included studies | | | | | | | | | |
| --- | --- | --- | --- | --- | --- | --- | --- | --- | --- |
| **Newcastle-Ottawa Scale for assessing the quality of studies in meta-analysis** | | | | | | | | | |
| **Study** | **SELECTION** | | | | **COMPARABILITY** | **OUTCOME** | | | **Scores** |
|  | **Representativeness of the Exposed Cohort** | **Selection of the Non-Exposed Cohort** | **Ascertainment of Exposure** | **Demonstration That Outcome of Interest Was Not Present at Start of Study** | **Comparability of Cohorts on the Basis of the Design or Analysis** | **Assessment of Outcome** | **Was Follow-Up Long Enough for Outcomes to Occur** | **Adequacy of Follow Up of Cohorts** |  |
| **Seeger** | ＊ | ＊ | ＊ | ＊ | ＊ | ＊ | ＊ | ＊ | **8** |
| **Polzin** | ＊ | ＊ | ＊ | ＊ | ＊ | ＊ | ＊ | ＊ | **8** |
| **Hohmann** | ＊ | ＊ | ＊ |  | ＊ | ＊ | ＊ | ＊ | **7** |
| **Cammalleri** | ＊ | ＊ | ＊ | ＊ | ＊ | ＊ | ＊ | ＊ | **8** |
| **Geis** | ＊ | ＊ | ＊ |  | ＊＊ | ＊ | ＊ | ＊ | **8** |
