# Supplementary material for: Anticoagulation therapy and clinical outcomes following transcatheter mitral valve repair for patients with mitral regurgitation: A meta‐analysis
Source: Clin Cardiol. 2023 Apr 10;46(6):598–606. doi: 10.1002/clc.24017 (PMC10270264; doi:10.1002/clc.24017)
Supplement: Supplementary file 14 — Supporting information. [file CLC-46-598-s012.docx]

| Outcomes of included Studies | | | | | | | | | | | | | | | | |
| --- | --- | --- | --- | --- | --- | --- | --- | --- | --- | --- | --- | --- | --- | --- | --- | --- |
| **Study** | **Intervention group** | | **Control group** | | **Intervention group** | | **Control group** | | **Intervention group** | | **Control group** | | **Intervention group** | | **Control group** | |
|  | **Bleeding （n）** | **No bleeding （n）** | **Bleeding （n）** | **No bleeding （n）** | **Stroke （n）** | **No stroke （n）** | **Stroke （n）** | **No stroke （n）** | **Combined endpoints （n）** | **No combined endpoints （n）** | **Combined endpoints （n）** | **No combined endpoints （n）** | **All-cause death （n）** | **No all-cause death （n）** | **All-cause death （n）** | **No all-cause death （n）** |
| **Seeger** | 0 | 136 | 0 | 118 | 0 | 136 | 1 | 117 | 1 | 135 | 5 | 113 | 1 | 135 | 4 | 114 |
| **Polzin** | 17 | 24 | 10 | 22 | 0 | 41 | 2 | 30 | N/A | N/A | N/A | N/A | N/A | N/A | N/A | N/A |
| **Hohmann** | 8 | 569 | 16 | 442 | N/A | N/A | N/A | N/A | N/A | N/A | N/A | N/A | 37 | 520 | 31 | 427 |
| **Cammalleri** | 2 | 19 | 5 | 34 | 0 | 21 | 0 | 39 | 5 | 16 | 10 | 29 | 3 | 18 | 5 | 34 |
| **Geis** | 6 | 428 | 0 | 36 | 0 | 434 | 1 | 35 | N/A | N/A | N/A | N/A | N/A | N/A | N/A | N/A |
