# Supplementary material for: Anticoagulation therapy and clinical outcomes following transcatheter mitral valve repair for patients with mitral regurgitation: A meta‐analysis
Source: Clin Cardiol. 2023 Apr 10;46(6):598–606. doi: 10.1002/clc.24017 (PMC10270264; doi:10.1002/clc.24017)
Supplement: Supplementary file 15 — Supporting information. [file CLC-46-598-s002.docx]

Supplementary Table. 1 Quality assessment of included studies.

Supplementary Table. 2 Outcomes of included Studies.

Supplementary Figure 1. Subgroups analysis of OAC, OAC plus SAPT, and OAC plus DAPT vs. the non-anticoagulated group for bleeding (supplementary Figure 1.A), stroke (supplementary Figure 1.B), combined endpoints (supplementary Figure 1.C) and all-cause death (supplementary Figure 1.D).

Supplementary Figure 2. Sensitivity analyses for bleeding (supplementary Figure 2.A), stroke (supplementary Figure 2.B), combined endpoints (supplementary Figure 2.C) and all-cause death (supplementary Figure 2.D).

Supplementary Figure 3. Funnel plots for bleeding (supplementary Figure 3.A), stroke (supplementary Figure 3.B), combined endpoints (supplementary Figure 3.C) and all-cause death (supplementary Figure 3.D).
